# Postoperative Apical Ballooning Syndrome Following Orthotopic Liver Transplantation

**DOI:** 10.7759/cureus.34450

**Published:** 2023-01-31

**Authors:** Osamah Z Badwan, Osama Abu-Shawer, Aritra Paul, Michael Faulx, Benico Barzilai

**Affiliations:** 1 Internal Medicine, Cleveland Clinic, Cleveland, USA; 2 Surgery, Nil Ratan Sircar Medical College and Hospital, Kolkata, IND; 3 Cardiology, Cleveland Clinic, Cleveland, USA; 4 Heart, Vascular, and Thoracic Institute, Cleveland Clinic, USA

**Keywords:** post-op complications, cardiomyopathy, orthotropic liver transplant, takotsubo cardiomyopathy (ttc), apical ballooning syndrome

## Abstract

As the mainstay of therapy for end-stage liver disease (ESLD), orthotopic liver transplantation (OLT) has complex effects on multiple organ systems. We present a representative case of acute heart failure with apical ballooning syndrome following OLT and review its mechanisms. Recognition of this and other potential cardiovascular and hemodynamic complications of OLT are essential to periprocedural anesthesia management. Once an acute phase of the condition is stabilized, conservative treatment and resolution of physical or emotional stressors usually allow for rapid resolution of symptoms, typically recovering systolic ventricular function within one to three weeks.

## Introduction

End-stage liver disease (ESLD) carries a grim prognosis with a 50% mortality rate at one year without transplant [1]. Orthotopic liver transplantation (OLT) is the mainstay therapy for this disease, with a five-year survival rate of 75% [2]. However, the OLT procedure is a complex procedure and may result in several intraoperative and postoperative complications. Here, we describe a case of acute heart failure in the setting of apical ballooning syndrome (ABS) following the OLT procedure.

## Case presentation

A 52-year-old woman was admitted for liver transplantation. She had end-stage liver disease with a Model for End-Stage Liver Disease (MELD) score of 21 due to cirrhosis caused by chronic hepatitis C virus (HCV) infection and alpha-1-antitrypsin (AAT) deficiency that was also complicated by hepatocellular cancer, for which the patient underwent radiofrequency ablation one year prior to the liver transplant. The patient also had a generalized anxiety disorder (GAD), asthma, AAT-related lung disease, and obesity class II for which the patient had undergone a laparoscopic sleeve gastrectomy in 2016. She had undergone extensive pre-transplant workup including echocardiography that revealed a normal left ventricular ejection fraction (LVEF) of 65% and a dobutamine stress echocardiogram that showed no evidence of coronary ischemia at 90% of the maximum predicted heart rate. The patient underwent OLT under general anesthesia with routine endotracheal intubation, invasive arterial pressure, pulmonary artery catheter, and thromboelastography monitoring. Intraoperatively, she lost about three liters of blood and was given eight units of packed red blood cells, two units of platelets, two units of cryoprecipitate, and a unit of fresh frozen plasma, consistent with a routine OLT.

Following the surgery, the patient was admitted to the surgical intensive care unit for close monitoring. The following day, she developed sinus tachycardia and postoperative hypotension (blood pressure: 80/40mmHg), which warranted starting vasopressor infusion (4.5mL/hour norepinephrine) to maintain the blood pressure >100/60mmHg. The shock was multifactorial (hypovolemic and vasodilatory) in the setting of low-volume resuscitation and the use of anesthetics/muscle relaxants perioperatively. The blood culture was negative, chest radiography was unremarkable and there were no signs of cardiogenic shock. However, the patient was also started on several antimicrobial agents as well as a routine transplant immunosuppressive regimen (mycophenolate mofetil, tacrolimus, and prednisone). She eventually improved during the following days, was weaned from all pressors, and transferred to the regular nursing floor.

**Figure 1:**
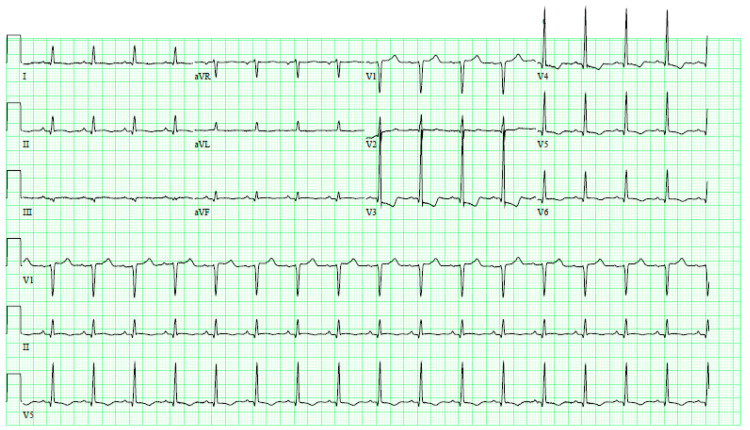
EKG showing normal sinus rhythm with nonspecific ST segment changes EKG: electrocardiogram

On the sixth postoperative day, the patient reported dyspnea, orthopnea, and paroxysmal nocturnal dyspnea, but denied any chest pain or palpitations. The physical exam showed bilateral pitting edema, bibasilar crackles, and normal heart sounds with no murmurs upon auscultation. Initial workup showed elevated NT-pro B-type natriuretic peptide at 20,405 pg/mL, troponin T of 0.152ng/mL (peak value), and normal sinus rhythm with nonspecific ST-segment changes on the electrocardiogram (EKG), (Figure 1). Her InterTAK diagnostic score was 73, representing a 91.7% probability of ABS based on the Takotsubo International Registry [3].

**Figure 2:**
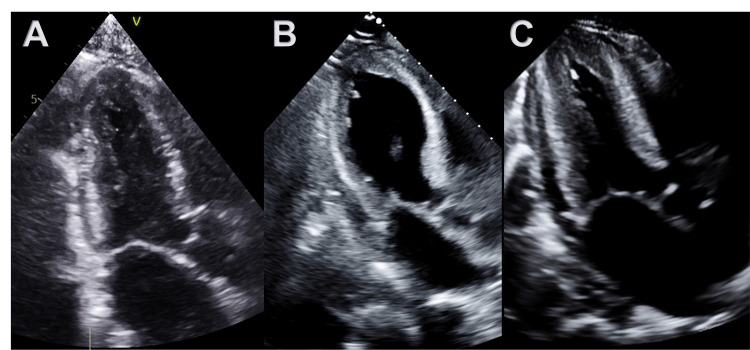
Echocardiography images (apical 3-chamber view) (A) Normal apical systole four months pre-op, (B) mid and distal anterior septum and entire apex are akinetic with preserved contraction in basal portion at six days post-OLT, (C) Recovered LV function with normal apical systole 20 days post-OLT OLT: orthotopic liver transplantation; LV: left ventricle

**Figure 3:**
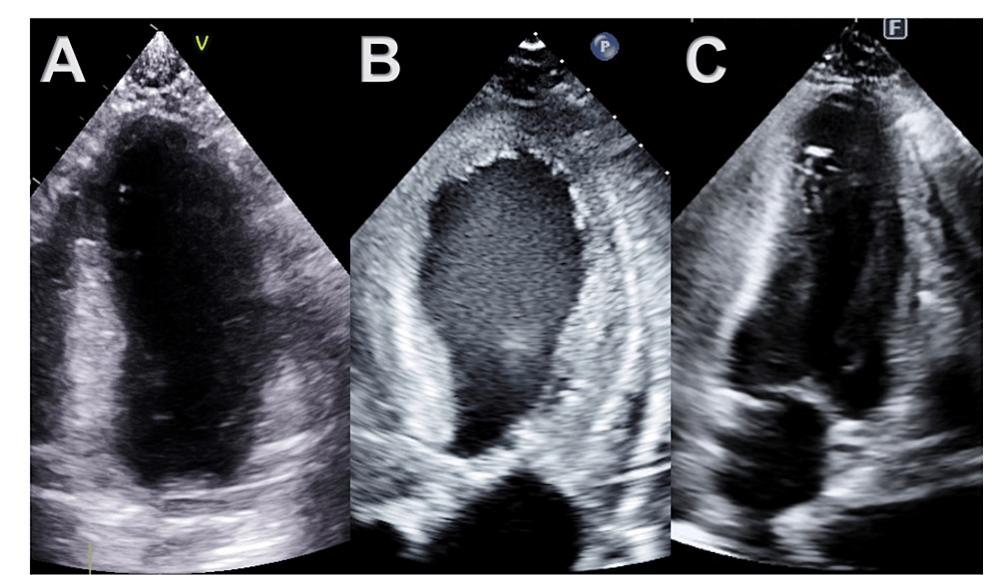
Echocardiography images (apical 2-chamber views) (A) Normal apical systole 4 months pre-op, (B) mid and distal anterior septum and entire apex are akinetic with preserved contraction in basal portion at six days post-OLT, (C) Recovered LV function with normal apical systole 20 days post-OLT OLT: orthotopic liver transplantation; LV: left ventricle

Given these significant changes in the patient’s clinical status, echocardiography was obtained which showed a significant drop in the LVEF from 65% to 35% with resting wall motion abnormalities, manifested as an akinetic mid and distal anterior septum, severely hypokinetic mid anterior segment, and mildly hypokinetic mid inferolateral, inferolateral, inferoseptal, and inferior segments forming apical ballooning and basal hypercontractility consistent with the typical picture of ABS syndrome (Video 1), with no evidence of left ventricular outflow tract obstruction (Figures 2, 3). A coronary angiography study was done which showed no evidence of coronary artery disease. We also continued monitoring the troponins, which was down-trending. Angiotensin-converting enzyme inhibition was started, given a limited body of evidence supporting its use for left ventricular systolic dysfunction in patients with ABS. A follow-up echocardiogram at 20 days post-OLT demonstrated a recovered LVEF at 57% with no wall motion abnormalities (Figures 2, 3) in addition to her resolution of symptoms.

**Video 1:** Echocardiography images (apical 4-chamber view) Resting wall motion abnormalities, manifested as akinetic mid and distal anterior septum, severely hypokinetic mid anterior segment, and mildly hypokinetic mid inferolateral, inferolateral, inferoseptal, and inferior segments forming apical ballooning and basal hypercontractility.

## Discussion

At our center, we perform over 200 orthotopic liver transplants annually, however, we rarely encounter postoperative ABS complications. Here, we report a case of ABS following liver transplant surgery. In our reported case, the diagnosis of ABS was made based on Mayo's criteria after the coronary angiography showed no evidence of coronary artery disease and the troponin level was down-trending [4]. The patient was started then on diuretics (furosemide), angiotensin-converting enzyme inhibitors, and inotropic support briefly for 24 hours with norepinephrine and responded well to the medical regimen. The patient did not need temporary mechanical circulatory support which is sometimes needed as a rescue measurement for patients with severe ABS.

Idiopathic ABS, also known as Takotsubo Cardiomyopathy, is a reversible cause of acute heart failure, precipitated by an emotional, chemical, or physical stressor [5]. The typical variant of ABS is characterized by mid and apical left ventricle (LV) segments hypokinesis in the absence of any evidence of coronary artery ischemia along with preserved basal LV segment’s function manifested classically as apical ballooning, resembling the takotsubo pot, a pot historically used to catch the octopuses in Japan. ABS has been reported following major surgical procedures that pose significant stress on the heart [6-7]. It may present with chest pain, dyspnea, palpitations, and peripheral edema and might be severe enough to result in cardiogenic shock. The EKG could show ST elevations mimicking acute myocardial infarct or non-specific ST changes. 

Any significant emotional or physical stress could trigger a catecholamine surge that can lead to capillaries spasms with a marked increase in the afterload via the beta-adrenergic receptors, which in turn results in LV systolic dysfunction, manifested as ABS [5]. Liver transplant surgery is a tremendous physical and emotional stressor that is associated with a dramatic increase in the concentrations of catecholamines perioperatively, which could contribute to the development of ABS [8]. Although ABS is considered a self-limited condition, it might result in consequential morbidity and mortality in patients with multiple comorbidities with a mortality rate of around 4% [4]. Patients with advanced-stage liver disease have an inadequate chronotropic and ionotropic response to stress as a sequela of what is known as cirrhotic cardiomyopathy [9]. This disturbed cardiac function is likely mediated by beta-adrenergic receptor dysfunction, increased cardio-depressant inflammatory mediators, and delayed repolarization [9].

## Conclusions

Perioperative stress cardiomyopathy or ABS diagnosis should be considered in OLT patients who developed heart failure symptoms and signs after surgery, especially in patients with high MELD scores. The approach for managing ABS post liver transplantation is not well defined and is usually based on supportive therapy. However, diuretics and inotropic/vasopressor support are the standard approaches for managing acute LV dysfunction, with circulatory support devices as second-line management if the former strategies fail. Conservative treatment and resolution of physical or emotional stressors usually allow for rapid resolution of symptoms, typically recovering systolic ventricular function within one to three weeks, highlighting the importance of recognition of ABS as a potential post-OLT complication given the higher rates of in-hospital complications.
